# Emerging Catatonia and Psychosis in Resolving COVID-19 Infection in an Adolescent

**DOI:** 10.1192/j.eurpsy.2022.1259

**Published:** 2022-09-01

**Authors:** N. Choudhury, B. Milo, P. Chen, B. Carr

**Affiliations:** 1 Nova Southeastern University, Dr. Kiran C. Patel College Of Allopathic Medicine, Davie, United States of America; 2 Nova Southeastern University, Kiran C. Patel College Of Osteopathic Medicine, Hallandale, United States of America; 3 University of Florida, Psychiatry, Gainesville, United States of America

**Keywords:** Covid-19, Psychosis, ECT, Catatonia

## Abstract

**Introduction:**

COVID-19 infection may lead to encephalopathy and various neurotrophic effects which can result in neuropsychiatric complications. Here, an asymptomatic adolescent female developed acute onset catatonia and psychosis manifesting during the resolution of Covid-19 infection.

**Objectives:**

Discuss differential diagnosis, medical workup, and initial treatment optimization for acute stabilization.

**Methods:**

This 15-year-old female with no previous psychiatric history nor prodromal symptomatology was hospitalized secondary to Covid -19. During the immediate three-month recovery phase following resolution of Covid-19, the patient exhibited gradually increasing anxiety, paranoia, delusions, disorganized behavior, and weight loss leading to re-hospitalization secondary to catatonia. Negative workup included rapid strep test, urinalysis, chest and abdominal x-ray, EEG, and brain MRI. Lumbar puncture revealed elevated WBC of 18 but was unremarkable for NDMA receptor antibodies, CSF HSV, and encephalitis panel. IV steroids, IVIG, and Anakinra were all given without benefit. Inadequate response to olanzapine, clonidine, and lorazepam led to an Index Series of bilateral electroconvulsive therapy (ECT).

**Results:**

The provisional diagnosis of psychotic disorder secondary to COVID-19 infection responded robustly regarding sleep, behavior, and affect by session #6, yet positive symptoms of psychosis persist. Ongoing ECT, psychopharmacology, and narrowing of the differential diagnosis continue.

**Conclusions:**

As more COVID-19 cases evolve during the pandemic, potential post-infectious neuropsychiatric complications should be considered as potentially contributory and kept in a thoughtful differential diagnosis. Regardless of ultimate causation, the acute symptom profile responded robustly to an initial Index Series of ECT.

**Disclosure:**

No significant relationships.

